# Prevalence of anxiety and associated factors among guardians of children admitted with severe malaria at Thyolo District Hospital in Malawi

**DOI:** 10.4102/sajpsychiatry.v31i0.2591

**Published:** 2025-12-17

**Authors:** Nixon Chidzere, Chimwemwe Munthali, George Chapweteka, Thandie Munthali, Patson Kumwenda, Blessings Chikasema, Esmie Mkwinda, Geldine Chironda

**Affiliations:** 1 Department of Clinical Medicine, Faculty of Health Sciences, Saint John of God University, Mzuzu, Malawi, Malawi; 2 Department of Nursing and Midwifery, Faculty of Health Sciences, Saint John of God University, Mzuzu, Malawi; 3 Seed Global Health, Lilongwe, Malawi

**Keywords:** severe malaria, guardians, anxiety, caregivers, hospital admission, mental health, Malawi

## Abstract

**Background:**

Anxiety is a common psychological response among caregivers of children with severe illnesses, yet limited research has explored its prevalence and associated factors in guardians of children with severe malaria in Malawi. Understanding the severity of anxiety and its correlates is crucial for supporting caregiver mental health and improving paediatric outcomes.

**Aim:**

This study aimed to determine the prevalence and severity of anxiety, and identify social, demographic and clinical factors associated with anxiety among guardians of children admitted with severe malaria.

**Setting:**

The study setting was the Peadiatric ward of Thyolo District Hospital in Malawi.

**Methods:**

A cross-sectional study was conducted involving 187 guardians of children diagnosed with severe malaria. Data were collected using a structured questionnaire incorporating socio-demographic and clinical information, alongside the Generalised Anxiety Disorder 7-item (GAD-7) scale. Descriptive statistics, Chi-square tests and logistic regression were used to analyse the data.

**Results:**

Mild anxiety was the most common (79.7%) among participants, followed by moderate (10.7%) and minimal (9.6%), with no cases of severe anxiety. Significant socio-demographic factors associated with higher anxiety included age (*p* < 0.001), guardian type (*p* < 0.001), education level (*p* < 0.001), area of residence (*p* < 0.001) and source of income (*p* < 0.001). Clinically, longer hospital stays (*p* < 0.001) and repeated admissions (*p* < 0.001) were significantly associated with increased anxiety severity.

**Conclusion:**

Anxiety is prevalent among guardians of children with severe malaria, with several socio-demographic and clinical factors contributing to its severity. The findings highlight the importance of integrating psychological support services into paediatric care settings.

**Contribution:**

This study underscores the need for routine anxiety screening, mental health support and context-specific interventions targeting caregivers of hospitalised children with severe illnesses in low-resource settings.

## Introduction

Malaria remains a major public health concern in sub-Saharan Africa, including Malawi, contributing significantly to morbidity and mortality, particularly among children under five and pregnant mothers.^1^ When a child is admitted, guardians often experience intense stress, leading to high anxiety levels.^2^ Guardians in the context of paediatric healthcare are individuals who play a vital role in caring for hospitalised children.^3^ These guardians may include family members, parents, legal custodians or close caregivers entrusted with the child’s well-being. In the hospital setting, these guardians often serve as the child’s primary advocates, offering emotional support and actively participating in the decision-making process regarding the child’s care.^4,5^

Guardians are typically encouraged to be present with the hospitalised child throughout their hospital stay.^6^ The presence of guardians is crucial for providing comfort and reassurance to the children, assisting with daily activities and ensuring effective communication between healthcare providers and the child.^7^ However, guardians’ level of involvement and availability may vary based on individual circumstances, cultural norms and healthcare or hospital policies.^5^ Thus, the hospitalisation of a child is a challenging and emotionally intense experience for guardians who are responsible for that child’s care.

The emotional strain can progress into anxiety, which significantly impacts both caregivers’ well-being and the quality of care provided to the child.^8^ Paediatric guardians often experience heightened anxiety levels, stemming from a variety of factors, which include but are not limited to duration of hospitalisation, and type of treatment, the unfamiliar hospital environment, concerns about the child’s treatment and recovery and the emotional toll of witnessing their young loved one in distress.^9,10^ The anxiety experienced by guardians can profoundly impact the quality of care provided to the child and their own well-being.^11^

The prevalence of severe malaria among children in sub-Saharan Africa is a significant public health concern, leading to high rates of morbidity and mortality.^1^ In Malawi, malaria is the leading cause of paediatric admissions, with around 40% of cases being attributed to this disease.^12^ In Malawi’s healthcare system, guardians are vital for hospitalised children’s care, offering emotional support, aiding with daily tasks and working closely with healthcare staff in treatment decisions.^13^ However, there are limited data specifically addressing the prevalence and associated factors of anxiety among guardians of children admitted with severe malaria in Malawi. Therefore, this research investigated the prevalence of anxiety and associated factors among guardians of children admitted with severe malaria at Thyolo District Hospital in Malawi.

## Research methods and design

### Study design and setting

The study employed a descriptive cross-sectional quantitative design. This study was conducted at Thyolo District Hospital paediatric ward, which is one of the district hospitals in the southern region of Malawi, from April 2024 to June 2024 had a total of 1501 admissions, including 709 severe malaria cases.

### Study population and sampling strategy

In this research, the population consisted of guardians whose children were admitted to the paediatric ward with a diagnosis of severe malaria and started on antimalarial treatment. The diagnosis of severe malaria was based on clinical assessment rather than laboratory confirmation. Inclusion criteria include guardians aged 18 years or older who have been with a child diagnosed with severe malaria in the paediatric ward for a minimum of 12 h. The exclusion criteria include guardians with any cognitive impairments and those whose children were diagnosed with conditions other than severe malaria. Using an average of 260 monthly severe malaria admissions from Thyolo District Hospital’s Health Management Information System, the sample size for the study was calculated with the Yamane formula at a 95% confidence level and 5% margin of error,^14^ resulting in a final sample size of 187 participants.

### Data collection

In this study, data were gathered using a structured questionnaire comprising two main sections: socio-demographic information and clinical characteristics. The socio-demographic section covered variables such as age, sex, economic status, area of residence, education level and duration of hospital stay. The clinical section included symptoms related to severe malaria and the frequency of hospital admissions, excluding anxiety. To assess anxiety levels, the Generalised Anxiety Disorder 7-item (GAD-7) scale was employed, which contains seven items focused on anxiety symptoms.^15^

The GAD-7 is a globally recognised screening tool for generalised anxiety disorder, with established cut-off scores of 5, 10 and 15 indicating mild, moderate and severe anxiety, respectively. This tool was used in this study instead of the State-Trait Anxiety Inventory (STAI)^16^ because it is a brief, reliable and validated tool suitable for assessing current anxiety symptoms within a short time frame, aligning with the study’s cross-sectional design. Unlike the STAI, which is lengthy and measures both long-term and temporary anxiety, the GAD-7 focuses on recent symptoms over the past 2 weeks, making it more practical for use in busy, resource-limited hospital settings such as Thyolo District Hospital.

Although the tool has not been formally validated in Malawi, it has demonstrated strong reliability and validity in several African countries, including Zimbabwe, where it showed good sensitivity (89%) and specificity (73%) along with a high internal consistency (Cronbach’s alpha of 0.87).^17^ In addition, the GAD-7 has been utilised in previous mental health studies conducted in Malawi, suggesting its practical applicability in the local context.^18^ Data collection was carried out through face-to-face interviews with guardians at Thyolo District Hospital, using the GAD-7 questionnaire. Each interview lasted approximately 10 min, during which participants responded to the questionnaire items while the researcher recorded their answers.

### Ethical considerations

An application for full ethical approval was made to the Mzuzu University Faculty of Health Sciences Research and Ethics Committee and the Saint John of God College of Health Sciences Research and Ethics Committee, and ethical consent was received on 30 October 2024. The ethical approval number (FOHS/STJOG/24/002). All procedures performed in this study involving human participants were in accordance with the ethical standards of the institution and the 1964 Helsinki Declaration of global ethical standards.^19^ Informed written consent was secured from each participant after the study’s purpose and potential benefits were clearly explained. Participants were also informed about their anonymity, the confidentiality of their information, and their right to participate voluntarily or withdraw from the study at any time.

## Results

### Socio-demographic and clinical characteristics of the respondents

The study involved 187 participants, with a majority being female (96.3%) and only a small number being male (3.7%). Most participants were aged between 18 years and 50 years (87.2%), while 12.8% were over 50. In terms of residence, 62.0% lived in rural areas, 22.5% in urban areas and 15.5% in semi-urban areas. Farming was the main source of income for 58.3% of participants, followed by business (30.5%) and employment (11.2%). Educationally, the highest proportion had completed Standard 5 to 8 (35.3%), followed by Form 1 to 2 (33.7%), Form 3 to 4 (16.6%), Standard 1 to 4 (13.9%), and only one participant (0.5%) had never attended school (Table 1). Among the 187 participants, the majority were mothers of the sick children (73.8%), while 13.9% were grandmothers and 10.7% were fathers. Clinically, the children of participating guardians exhibited severe malaria symptoms such as convulsions in 64.2% of cases, anaemia in 23.5%, and diarrhoea or vomiting in 10.7%. Most guardians reported that their children stayed in the ward for 2 days (67.4%), followed by 3 days (29.9%), and only a few stayed for more than 3 days (2.7%) (see Table 2).

**TABLE 1:** Social demographics and clinical characteristics (*N* = 187).

Variable	Frequency	%
**Socio-demographic characteristics**		
**Gender**		
Female	180	96.3
Male	7	3.7
**Age (years)**		
18–50	163	87.2
50 >	24	12.8
**Source of income**		
Employed	21	11.2
Business	57	30.5
Farmer	109	58.3
**Education background**		
Not attended education	1	0.5
Standard 1 to 4	26	13.9
Standard 5 to 8	66	35.3
Form 1 to 2	63	33.7
Form 3 to 4	31	16.6
**Area of residence**		
Urban	42	22.5
Semi-urban	29	15.5
Rural	116	62.0
**Type of guardian**		
Mother	138	73.8
Father	20	10.7
Grand mother	26	13.9
Uncle	1	0.5
Aunt	1	0.5
Sister	1	0.5
**Clinical characteristics**		
**Number of days in the ward**		
2	126	67.4
3	56	29.9
More than 3	5	2.7
**Severe malaria presentation**		
Convulsion	120	64.2
Anaemia	44	23.5
Diarrhoea and Vomiting	20	10.7
Others	3	1.6

**TABLE 2:** Prevalence and level of anxiety (*N* = 187).

Anxiety Levels	Frequency	%
Minimal anxiety	18	9.6
Mild anxiety	149	79.7
Moderate anxiety	20	10.7

**Total**	**187**	**100.0**

### Prevalence and levels of anxiety among guardians of children admitted with severe malaria

The study found that the majority of guardians experience mild anxiety, with 79.7% of them reporting this level. A smaller proportion, 10.7%, experience moderate anxiety, while 9.6% report minimal anxiety and no participant was found with a severe anxiety level (see Table 2).

### Factors associated with anxiety of guardians

The analysis reveals several significant factors associated with anxiety severity, as determined by Chi-square tests. Significant socio-demographic factors associated with higher anxiety included age (*p* < 0.001), guardian type (*p* < 0.001), education level (*p* < 0.001), area of residence (*p* < 0.001) and source of income (*p* < 0.001). Clinically, longer hospital stays (*p* < 0.001) and repeated admissions (*p* < 0.001) were significantly associated with increased anxiety severity (see Table 3).

**TABLE 3:** Social demographic and clinical factors associated with anxiety.

Variable	Anxiety level	*n*	%	*p*-value
**Gender**	-	-	-	0.614
Female	Minimal	17	9.4	-
	Mild	143	79.4	-
	Moderate	20	11.1	-
Male	Minimal	1	14.3	-
	Mild	6	85.7	-
**Age (years)**	-	-	-	0.000
18–49	Minimal	18	11.0	-
	Mild	143	87.7	-
	Moderate	2	1.2	-
50 >	Mild	6	25.0	-
	Moderate	18	75.0	-
**Type of guardian**	-	-	-	0.000
Mother	Minimal	15	10.9	-
	Mild	122	88.4	-
	Moderate	1	14.8	-
Father	Minimal	1	5.0	-
	Mild	19	15.9	-
Grand mother	Minimal	0	0.0	-
	Mild	7	26.9	-
	Moderate	19	73.1	-
Uncle	Minimal	1	100.0	-
Aunt	Minimal	1	100.0	-
Sister	Mild	1	100.0	-
**Number of days**	-	-	-	0.000
2	Mild	1	100.0	-
3	Mild	41	73.2	-
	Moderate	15	26.8	-
3 >	Mild	5	100.0	-
**Severe malaria presentation**	-	-	-	0.000
Convulsions	Mild	103	85.8	-
	Moderate	17	14.1	-
Anaemia	Minimal	17	36.8	-
	Mild	24	54.5	-
	Moderate	3	6.8	-
Diarrhoea and vomiting	Mild	20	100.0	-
Others	Minimal	1	33.3	-
	Mild	2	66.6	-
**Area of residence**	-	-	-	0.000
Urban	Minimal	3	7.1	-
	Mild	39	92.8	-
Semi-Urban	Minimal	15	51.7	-
	Mild	14	48.2	-
Rural	Mild	96	82.7	-
	Moderate	20	17.2	-
**Education level**	-	-	-	0.000
Not attended education	Moderate	1	100.0	-
Standard 1 to 4	Mild	8	30.7	-
	Moderate	18	69.2	-
Standard 5 to 8	Mild	65	98.4	-
	Moderate	1	1.5	-
Form 1 to 2	Minimal	16	25.3	-
	Mild	47	74.6	-
Form 3 above	Minimal	2	6.4	
	Mild	29	93.5	

## Discussion

The study assessed anxiety levels among guardians of children admitted with severe malaria, focusing on demographic and clinical factors. The majority of guardians in this study reported experiencing mild anxiety, with only a small proportion experiencing moderate anxiety and minimal anxiety. Notably, no guardian reported severe anxiety. Thus, persistent mild anxiety can lead to stress-related health problems, such as emotional exhaustion and reduced physical well-being. Previous studies have shown that chronic low-level anxiety among caregivers is linked to long-term mental distress and diminished caregiving capacity.^20^ Additionally, the hormesis theory proposes that mild anxiety may sometimes have adaptive effects, helping individuals to remain alert, focused and effective under pressure.^21,22^ Thus, mild anxiety in guardians might represent a tolerable level of stress that enhances caregiving performance rather than hindering it.

This study’s finding suggests that while the emotional impact of caring for a child with severe malaria is substantial, most guardians are able to manage their anxiety at a mild level. However, the absence of severe anxiety might indicate that, despite the critical nature of the child’s condition, guardians may not reach the highest levels of anxiety. Similar studies have shown that anxiety for children with severe health conditions can lead to heightened anxiety, but severe levels of distress are less common, possibly because of the hospital and family support, which gives hope or perceived control over the child’s recovery.^23^ The relatively high prevalence of mild anxiety could be attributed to the inherent stress of caregiving in a hospital setting, especially when the child’s condition is severe, but not life-threatening, to the point of overwhelming the guardian’s ability to function. However, some research suggests that even mild anxiety can accumulate and affect the caregiver’s long-term well-being.^24^

The social demographic characteristics significantly associated with anxiety severity, as determined by chi-square tests, include several critical factors. Age emerged as a strong determinant (*p* < 0.001), with older individuals (50 years and above) experiencing higher levels of anxiety compared to younger groups, this is in line with a study by Canuto et al.,^25^ suggesting that older guardians might face increased stress because of factors, such as physical challenges, less flexibility in caregiving roles or concerns about long-term health outcomes for their children.

The type of guardian also showed a significant association (*p* < 0.001); grandmothers demonstrated greater anxiety severity than other guardians. This is in line with existing literature, which suggests that women may experience higher levels of anxiety because of caregiving roles.^26^ Education level was another significant factor (*p* < 0.001); lower educational attainment was linked to moderate anxiety. This finding suggests that lower levels of education might be associated with less access to health information,^27^ or greater difficulty in navigating the healthcare system,^28^ all of which could increase stress levels. Similarly, the area of residence played a role (*p* < 0.001), with rural residents more prone to higher anxiety levels than urban or semi-urban dwellers. Additionally, source of income exhibited a strong association (*p* < 0.001); farmers were more likely to report moderate anxiety compared to business owners or formally employed individuals. This could be because of various factors, including limited access to healthcare resources, transportation difficulties or a lack of immediate social support networks in rural areas.^29^

Clinically, children presented with a range of severe malaria symptoms, including convulsions, anaemia and diarrhoea and vomiting. Despite these severe presentations, no clear patterns emerged between specific clinical manifestations and the anxiety levels of guardians. This could imply that the guardians’ anxiety is more closely related to the overall experience of caregiving in a hospital setting rather than specific symptoms. Similar findings have been observed in other studies, where the stress experienced by caregivers often stems from the uncertainty of the child’s prognosis, rather than a specific clinical event.^23^ Furthermore, the duration of hospital stay was found to have a statistically significant relationship (*p* < 0.001) with anxiety severity. Patients staying for 2 days mostly experienced minimal to mild anxiety, while those staying for 3 days or more showed increased anxiety levels, with many moving into the moderate anxiety category. Thus, prolonged hospital stays may increase anxiety because of prolonged exposure to a stressful environment and uncertainty about recovery.^30^

The analysis reveals a strong and statistically significant association between the number of hospital admissions and anxiety severity (*p* < 0.001). Patients with only one admission predominantly experienced minimal anxiety, indicating that initial hospital stays may not substantially elevate anxiety levels. However, those with two admissions showed a sharp increase in mild anxiety cases, while guardians with three or more admissions exhibited higher rates of moderate anxiety. This trend suggests that repeated hospitalisations progressively worsen anxiety levels,^31^ highlighting the need for comprehensive psychological support.

### Limitations of the study

This study’s limitations include its cross-sectional design, which prevents causal inference, reliance on self-reported data that may introduce bias, and its single-site nature, which limits generalisability. Additionally, the GAD-7 may not fully capture situational or trait-specific anxiety common in caregiving contexts.^32^

## Conclusion

Overall, the findings of this study reveal significant associations between both socio-demographic and clinical factors and anxiety severity among patients. The study also points to the need for additional support mechanisms for guardians of children hospitalised because of severe malaria, particularly those of advanced age, women, guardians from rural areas, those with lower educational attainment, guardians who have stayed with a child for a long time in the hospital and guardians with a child admitted several times. Future studies using longitudinal or mixed methods should explore whether mild anxiety functions as a protective or harmful factor for caregivers over time to help clarify causal relationships and inform interventions for caregiver mental health. Moreover, research with larger and more diverse samples and additional qualitative methods could provide deeper insights into the complex interplay between demographic, clinical and psychological factors affecting caregivers of children with severe malaria.
